# Non-contiguous finished genome sequence of the opportunistic oral pathogen *Prevotella multisaccharivorax* type strain (PPPA20^T^)

**DOI:** 10.4056/sigs.2164949

**Published:** 2011-09-23

**Authors:** Amrita Pati, Sabine Gronow, Megan Lu, Alla Lapidus, Matt Nolan, Susan Lucas, Nancy Hammon, Shweta Deshpande, Jan-Fang Cheng, Roxanne Tapia, Cliff Han, Lynne Goodwin, Sam Pitluck, Konstantinos Liolios, Ioanna Pagani, Konstantinos Mavromatis, Natalia Mikhailova, Marcel Huntemann, Amy Chen, Krishna Palaniappan, Miriam Land, Loren Hauser, John C. Detter, Evelyne-Marie Brambilla, Manfred Rohde, Markus Göker, Tanja Woyke, James Bristow, Jonathan A. Eisen, Victor Markowitz, Philip Hugenholtz, Nikos C. Kyrpides, Hans-Peter Klenk, Natalia Ivanova

**Affiliations:** 1 DOE Joint Genome Institute, Walnut Creek, California, USA; 2 DSMZ - German Collection of Microorganisms and Cell Cultures GmbH, Braunschweig, Germany; 3 Los Alamos National Laboratory, Bioscience Division, Los Alamos, New Mexico, USA; 4 Biological Data Management and Technology Center, Lawrence Berkeley National Laboratory, Berkeley, California, USA; 5 Oak Ridge National Laboratory, Oak Ridge, Tennessee, USA; 6 HZI – Helmholtz Centre for Infection Research, Braunschweig, Germany; 7 University of California Davis Genome Center, Davis, California, USA; 8 Australian Centre for Ecogenomics, School of Chemistry and Molecular Biosciences, The University of Queensland, Brisbane, Australia

**Keywords:** obligately anaerobic, non-motile, Gram-negative, mesophilic, chemoorganotrophic, opportunistic pathogen, *Prevotellaceae*, GEBA

## Abstract

*Prevotella multisaccharivorax* Sakamoto *et al.* 2005 is a species of the large genus *Prevotella*, which belongs to the family *Prevotellaceae*. The species is of medical interest because its members are able to cause diseases in the human oral cavity such as periodontitis, root caries and others. Although 77 *Prevotella* genomes have already been sequenced or are targeted for sequencing, this is only the second completed genome sequence of a type strain of a species within the genus *Prevotella* to be published. The 3,388,644 bp long genome is assembled in three non-contiguous contigs, harbors 2,876 protein-coding and 75 RNA genes and is a part of the *** G****enomic* *** E****ncyclopedia of* *** B****acteria and* *** A****rchaea * project.

## Introduction

Strain PPPA20^T^ (= DSM 17128 = JCM 12954) is the type strain of *Prevotella multisaccharivorax* [1]. Currently, there are about 50 species placed in the genus *Prevotella* [1]. The species epithet is derived from the Latin adjective *multus* meaning ‘many/much’, the Latin noun *saccharum* meaning ‘sugar’ and the Latin adjective *vorax* meaning ‘liking to eat’ referring to the metabolic properties of the species to digest a variety of carbohydrates [2]. *P. multisaccharivorax* strain PPPA20^T^ is considered to be an opportunistic pathogen and was isolated from subgingival plaque from a patient with chronic periodontitis. Additionally, five more strains isolated from the human oral cavity were placed in the species *P. multisaccharivorax* [2]. Using non-culture techniques on sites affected by endodontic and periodontal diseases, a large number of sequences have been found that belong to *Prevotella* and *Prevotella*-like bacteria. Many of those species have never been isolated or described [3]. The complex microbial community living in the rich ecological niche of the human oral cavity and its interaction with consumed food will be of lasting interest for medical and ecological reasons [4,5]. Here we present a summary classification and a set of features for *P. multisaccharivorax* PPPA20^T^, together with the description of the non-contiguous finished genomic sequencing and annotation.

## Classification and features

A representative genomic 16S rRNA sequence of *P. multisaccharivorax* PPPA20^T^ was compared using NCBI BLAST [6] under default settings (e.g., considering only the high-scoring segment pairs (HSPs) from the best 250 hits) with the most recent release of the Greengenes database [7] and the relative frequencies of taxa and keywords (reduced to their stem [8]) were determined, weighted by BLAST scores. The most frequently occurring genus was *Prevotella* (100.0%) (14 hits in total). Regarding the single hit to sequences from members of the species, the average identity within HSPs was 100.0%, whereas the average coverage by HSPs was 98.0%. Regarding the nine hits to sequences from other members of the genus, the average identity within HSPs was 90.3%, whereas the average coverage by HSPs was 66.5%. Among all other species, the one yielding the highest score was *Prevotella ruminicola* (AF218618), which corresponded to an identity of 91.5% and an HSP coverage of 66.3%. (Note that the Greengenes database uses the INSDC (= EMBL/NCBI/DDBJ) annotation, which is not an authoritative source for nomenclature or classification.) The highest-scoring environmental sequence was AY550995 ('human carious dentine clone IDR-CEC-0032'), which showed an identity of 99.8% and an HSP coverage of 94.5%. The most frequently occurring keywords within the labels of environmental samples which yielded hits were 'fecal' (4.4%), 'beef, cattl' (4.1%), 'anim, coli, escherichia, feedlot, habitat, marc, pen, primari, secondari, stec, synecolog' (4.0%), 'neg' (2.5%) and 'fece' (2.4%) (236 hits in total). The most frequently occurring keywords within the labels of environmental samples which yielded hits of a higher score than the highest scoring species were 'fece' (7.9%), 'goeldi, marmoset' (4.8%), 'microbiom' (4.3%), 'aspect, canal, oral, root' (3.9%) and 'rumen' (3.8%) (54 hits in total). While some of these keywords correspond to the well known habitat of *P. multisaccharivorax*, others indicate additional habitats related to animals.

**Figure 1:**
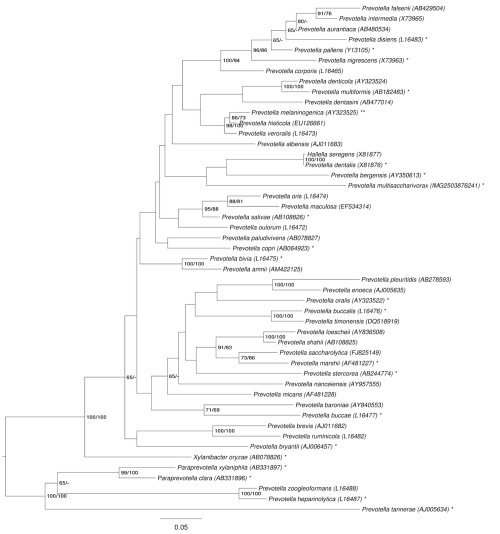
Phylogenetic tree highlighting the position of *P. multisaccharivorax* relative to the type strains of the other species within the family. The tree was inferred from 1,425 aligned characters [9,10] of the 16S rRNA gene sequence under the maximum likelihood (ML) criterion [11]. Rooting was done initially using the midpoint method [12] and then checked for its agreement with the current classification (Table 1). The branches are scaled in terms of the expected number of substitutions per site. Numbers adjacent to the branches are support values from 600 ML bootstrap replicates [13] (left) and from 1,000 maximum parsimony bootstrap replicates [14] (right) if larger than 60%. Lineages with type strain genome sequencing projects registered in GOLD [15] are labeled with one asterisk, those also listed as 'Complete and Published' should be labeled with two asterisks: *P. ruminicola* [16] and *P. melaninogenica* (CP002122/CP002123).

Figure 1 shows the phylogenetic neighborhood of *P. multisaccharivorax* in a 16S rRNA based tree. The sequences of the four 16S rRNA gene copies in the genome differ from each other by up to two nucleotides, and differ by up to two nucleotides from the previously published 16S rRNA sequence AB200414.

**Figure 2:**
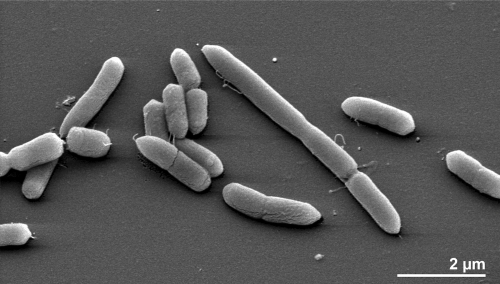
Scanning electron micrograph of *P. multisaccharivorax* PPPA20^T^

The cells of *P. multisaccharivorax* generally have the shape of rods (0.8 × 2.5-8.3 µm) and occur singly or in pairs (Figure 2). They can also form longer filaments. *P. multisaccharivorax* is a Gram-negative, non spore-forming bacterium (Table 1). The organism is described as non-motile and only four genes associated with motility were identified in the genome (see below). *P. multisaccharivorax* grows well at 37°C, is strictly anaerobic, chemoorganotrophic and is able to ferment cellobiose, glucose, glycerol, lactose, maltose, mannose, melezitose, raffinose, rhamnose, sorbitol, sucrose, trehalose and xylose [2]. Acid production from arabinose and salicin is variable. The organism does not reduce nitrate or produce indole from tryptophan but it hydrolyzes esculin and digests gelatin [2]. Growth of *P. multisaccharivorax* is inhibited by the addition of 20% bile. Major fermentation products are succinic and acetic acid, isovaleric acid is produced in small amounts [2]. Activities of glucose-6-phosphate dehydrogenase (G6PDH) and 6-phosphogluconate dehydrogenase (6GPDH) were not detected in isolates of this species, whereas malate dehydrogenase and glutamate dehydrogenase activities were detected in all strains. *P. multisaccharivorax* produces acid and alkaline phosphatase, β-galactosidase, α- and β-glucosidase, *N*-acetyl-β-glucosaminidase, α-aminofuranosidase and alanine aminopeptidase. The organism has no demonstrable esterase (C4), esterase lipase (C4), lipase (C4), leucine arylamidase, valine arylamidase, cystine arylamidase, pyroglutamic acid arylamidase, trypsin, chymotrypsin, β-glucuronidase, α-mannosidase, α-fucosidase, arginine aminopeptidase, leucine aminopeptidase, proline aminopeptidase, tyrosine aminopeptidase, phenylalanine aminopeptidase, urease or catalase activity [2].

**Table 1:** Classification and general features of *P. multisaccharivorax* PPPA20^T^ according the MIGS recommendations [17] and the NamesforLife database [1].

**MIGS ID**	**Property**	**Term**	**Evidence code**
	Current classification	Domain *Bacteria*	TAS [18]
		Phylum “*Bacteroidetes*”	TAS [19]
		Class “*Bacteroidia*”	TAS [20]
		Order “*Bacteroidales*”	TAS [21]
		Family “*Prevotellaceae*”	TAS [21]
		Genus *Prevotella*	TAS [22,23]
		Species *Prevotella multisaccharivorax*	TAS [2]
		Type strain PPPA20	TAS [2]
	Gram stain	negative	TAS [2]
	Cell shape	rod-shaped	TAS [2]
	Motility	non-motile	TAS [2]
	Sporulation	none	TAS [2]
	Temperature range	mesophilic	TAS [2]
	Optimum temperature	37°C	TAS [2]
	Salinity	physiological	TAS [2]
MIGS-22	Oxygen requirement	obligately anaerobic	TAS [2]
	Carbon source	carbohydrates	TAS [2]
	Energy metabolism	chemoorganotrophic	TAS [2]
MIGS-6	Habitat	host, human oral microflora	TAS [2]
MIGS-15	Biotic relationship	free-living	NAS
MIGS-14	Pathogenicity	opportunistic pathogen	TAS [2]
	Biosafety level	2	TAS [24]
	Isolation	subgingival plaque, chronic periodontitis	TAS [2]
MIGS-4	Geographic location	Japan	TAS [2]
MIGS-5	Sample collection time	December 9, 2002	IDA
MIGS-4.1	Latitude	not reported	
MIGS-4.2	Longitude	not reported	
MIGS-4.3	Depth	not reported	
MIGS-4.4	Altitude	not reported	

Evidence codes - IDA: Inferred from Direct Assay (first time in publication); TAS: Traceable Author Statement (i.e., a direct report exists in the literature); NAS: Non-traceable Author Statement (i.e., not directly observed for the living, isolated sample, but based on a generally accepted property for the species, or anecdotal evidence). These evidence codes are from of the Gene Ontology project [25]. If the evidence code is IDA, the property was directly observed by one of the authors or an expert mentioned in the acknowledgements.

### Chemotaxonomy

In contrast to other *Prevotella* species all strains of *P. multisaccharivorax* harbor the menaquinones MK-12 (40-55%) and MK-13 (40-45%) in large amounts, whereas MK-10 (1-3%) and MK-11 (8-10%) were found only in small amounts [2]. The fatty acid pattern for all strains of *P. multisaccharivorax* revealed C_18:1 ω9c_ (21.7%) and C_16:0_ (12.9%) as major compounds as well as *iso-*C_17:0 3-OH_ (9.2%), *anteiso*-C_15:0_ (7.8%), C_18:2 ω6,9c_ (7.5%) and *iso*-C_15:0_ (6.4%) in smaller amounts [2]. Additionally, the unusual dimethyl acetals were found with C_16:0_ dimethyl aldehyde in the highest amount of 8.2%. This clearly distinguishes the species of *P. multisaccharivorax* from other related *Prevotella* species [2].

## Genome sequencing and annotation

### Genome project history

This organism was selected for sequencing on the basis of its phylogenetic position [26], and is part of the *** G****enomic* *** E****ncyclopedia of* *** B****acteria and* *** A****rchaea * project [27]. The genome project is deposited in the Genomes On Line Database [15] and the complete genome sequence is deposited in GenBank. Sequencing, finishing and annotation were performed by the DOE Joint Genome Institute (JGI). A summary of the project information is shown in Table 2.

**Table 2:** Genome sequencing project information

**MIGS ID**	**Property**	**Term**
MIGS-31	Finishing quality	Non-contiguous finished
MIGS-28	Libraries used	Three genomic libraries: one 454 pyrosequence standard library, one 454 PE library (10 kb insert size), one Illumina library
MIGS-29	Sequencing platforms	Illumina GAii, 454 GS FLX Titanium
MIGS-31.2	Sequencing coverage	290.0 × Illumina; 48.0 × pyrosequence
MIGS-30	Assemblers	Newbler version 2.3, Velvet 0.7.63, phrap SPS 4.24
MIGS-32	Gene calling method	Prodigal 1.4, GenePRIMP
	INSDC ID	AFJE00000000 GL945015-GL945017
	Genbank Date of Release	June 20, 2011
	GOLD ID	Gi05358
	NCBI project ID	41513
	Database: IMG-GEBA	2503754046
MIGS-13	Source material identifier	DSM 17128
	Project relevance	Tree of Life, GEBA

### Growth conditions and DNA isolation

*P. multisaccharivorax* PPPA20^T^, DSM 17128, was grown anaerobically in DSMZ medium 104 (PYG-medium) [28] at 37°C. DNA was isolated from 0.5-1 g of cell paste using MasterPure Gram-positive DNA purification kit (Epicentre MGP04100) following the standard protocol as recommended by the manufacturer with modification st/DL for cell lysis as described in Wu *et al*. 2009 [27]. DNA is available through the DNA Bank Network [29].

### Genome sequencing and assembly

The genome was sequenced using a combination of Illumina and 454 sequencing platforms. All general aspects of library construction and sequencing can be found at the JGI website [30]. Pyrosequencing reads were assembled using the Newbler assembler (Roche). The initial Newbler assembly consisting of 154 contigs in five scaffolds was converted into a phrap [31] assembly by making fake reads from the consensus, to collect the read pairs in the 454 paired end library. Illumina GAii sequencing data (1,043.6 Mb) was assembled with Velvet [32] and the consensus sequences were shredded into 2.0 kb overlapped fake reads and assembled together with the 454 data. The 454 draft assembly was based on 135.4 Mb 454 standard data and all of the 454 paired end data. Newbler parameters are -consed -a 50 -l 350 -g -m -ml 20. The Phred/Phrap/Consed software package [31] was used for sequence assembly and quality assessment in the subsequent finishing process. After the shotgun stage, reads were assembled with parallel phrap (High Performance Software, LLC). Possible mis-assemblies were corrected with gapResolution [30], Dupfinisher [33], or sequencing cloned bridging PCR fragments with subcloning or transposon bombing (Epicentre Biotechnologies, Madison, WI). Gaps between contigs were closed by editing in Consed, by PCR and by Bubble PCR primer walks (J.-F. Chang, unpublished). A total of 218 additional reactions were necessary to close gaps and to raise the quality of the finished sequence. Illumina reads were also used to correct potential base errors and increase consensus quality using a software Polisher developed at JGI [34]. The error rate of the completed genome sequence is less than 1 in 100,000. Together, the combination of the Illumina and 454 sequencing platforms provided 338 × coverage of the genome. The final assembly contained 325,939 pyrosequence and 28,989,384 Illumina reads.

### Genome annotation

Genes were identified using Prodigal [35] as part of the Oak Ridge National Laboratory genome annotation pipeline, followed by a round of manual curation using the JGI GenePRIMP pipeline [36]. The predicted CDSs were translated and used to search the National Center for Biotechnology Information (NCBI) non-redundant database, UniProt, TIGR-Fam, Pfam, PRIAM, KEGG, COG, and InterPro databases. Additional gene prediction analysis and functional annotation was performed within the Integrated Microbial Genomes - Expert Review (IMG-ER) platform [37].

## Genome properties

**Figure 3:**
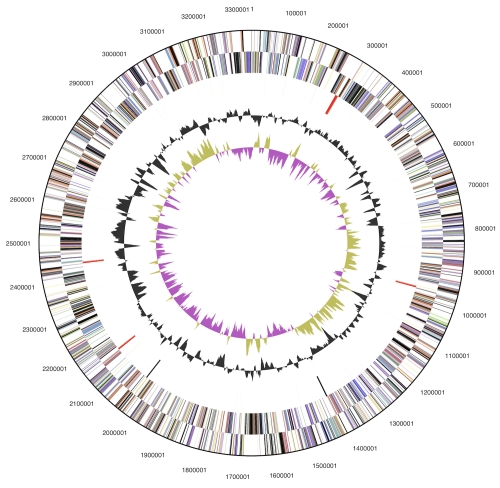
Graphical map of the largest scaffold. From outside to the center: Genes on forward strand (color by COG categories), Genes on reverse strand (color by COG categories), RNA genes (tRNAs green, rRNAs red, other RNAs black), GC content, GC skew.

The assembled genome sequence consists of three non-contiguous contigs with a length of 3,334,154 bp, 47,474 bp and 7,016 bp with a G+C content of 48.3% (Table 3 and Figure 3). Of the 2,951 genes predicted, 2,876 were protein-coding genes, and 75 RNAs; 166 pseudogenes were also identified. The majority of the protein-coding genes (60.5%) were assigned with a putative function while the remaining ones were annotated as hypothetical proteins. The distribution of genes into COGs functional categories is presented in Table 4.

**Table 3:** Genome Statistics

**Attribute**	**Value**	**% of Total**
Genome size (bp)	3,388,644	100.00%
DNA coding region (bp)	2,970,483	87.66%
DNA G+C content (bp)	1,636,375	48.31%
Number of scaffolds	3	
Total genes	2,951	100.00%
RNA genes	75	2.54%
rRNA operons	4-6	
Protein-coding genes	2,876	97.46%
Pseudo genes	166	5.63%
Genes in paralog clusters	438	14.84%
Genes assigned to COGs	1,659	56.22%
Genes assigned Pfam domains	1,864	63.17%
Genes with signal peptides	782	26.50%
Genes with transmembrane helices	588	19.93%
CRISPR repeats	3	

**Table 4:** Number of genes associated with the general COG functional categories

Code	value	%age	Description
J	138	7.7	Translation, ribosomal structure and biogenesis
A	0	0.0	RNA processing and modification
K	102	5.7	Transcription
L	183	10.1	Replication, recombination and repair
B	0	0.0	Chromatin structure and dynamics
D	26	1.4	Cell cycle control, cell division, chromosome partitioning
Y	0	0.0	Nuclear structure
V	46	2.6	Defense mechanisms
T	63	3.5	Signal transduction mechanisms
M	155	8.6	Cell wall/membrane/envelope biogenesis
N	4	0.2	Cell motility
Z	0	0.0	Cytoskeleton
W	0	0.0	Extracellular structures
U	31	1.7	Intracellular trafficking, secretion, and vesicular transport
O	69	3.8	Posttranslational modification, protein turnover, chaperones
C	90	5.0	Energy production and conversion
G	145	8.0	Carbohydrate transport and metabolism
E	132	7.3	Amino acid transport and metabolism
F	59	3.3	Nucleotide transport and metabolism
H	74	4.1	Coenzyme transport and metabolism
I	56	3.1	Lipid transport and metabolism
P	120	6.7	Inorganic ion transport and metabolism
Q	27	1.5	Secondary metabolites biosynthesis, transport and catabolism
R	202	11.2	General function prediction only
S	82	4.6	Function unknown
-	1,292	43.8	Not in COGs
